# *EHP* Paper of the Year, 2009

**DOI:** 10.1289/ehp.12903

**Published:** 2009-06

**Authors:** Hugh A. Tilson

**Affiliations:** Editor-in-Chief, *EHP,* E-mail: tilsonha@niehs.nih.gov

The Paper of the Year Award was established in 2008 by *Environmental Health Perspectives* (*EHP*) as a means of reinforcing high-quality articles published in the journal, identifying emerging research themes, and tracking the impact of groundbreaking research (Tilson 2008). In this issue, we are pleased to announce that the *EHP* Paper of the Year for 2009 is “Decrease in Anogenital Distance among Male Infants with Prenatal Phthalate Exposure” by Shanna H. Swan, Katharina M. Main, Fan Liu, Sara L. Stewart, Robin L. Kruse, Antonia M. Calafat, Catherine S. Mao, J. Bruce Redmon, Christine L. Ternand, Shannon Sullivan, J. Lynn Teague, and the Study for Future Families Research Team (Swan et al. 2005). We chose this paper because of its high impact in both the research and legislative realms since its publication in 2005.

This paper (Swan et al. 2005) was the first to demonstrate an association between pregnant women’s exposure to phthalates and adverse effects on genital development in their male children. Rodent studies had previously identified a syndrome of adverse effects of phthalates on the male reproductive system (Foster 2006; Sharpe 2005), and find-ings from Swan et al. (2005) supported the hypothesis that prenatal phthalate exposure at environmental levels can also adversely affect male reproductive development in humans. These findings are important because humans are commonly exposed to phthalates found in a wide variety of consumer products, including soft vinyl items, medical tubing and IV bags, and a variety of personal care products such as perfume, lotion, shampoo, cosmetics, nail polish, and hairspray.

Toxicologists routinely measure the external genitalia to assess reproductive toxicity in animal studies. One of these measures, anogenital distance (AGD)—a particularly sensitive indicator of masculinization—is shortened in male rodents following prenatal exposure to several phthalates (Foster 2006; Sharpe 2005). Swan et al. (2005) translated the standard animal exam to humans in order to investigate potential effects of phthalates on reproductive development in male infants. Specifically, they estimated associations between the presence and quantity of nine phthalate metabolites in mothers’ prenatal urine samples and AGD and other measurements in their sons. Higher levels of four phthalate metabolites [monoethyl phthalate (MEP), mono-*n*-butyl phthalate (MBP), monobenzyl phthalate (MBzP), and monoisobutyl phthalate (MiBP)] were associated with a shorter AGD. Swan subsequently replicated and extended these findings (2008).

Swan et al. (2005) has had an impact on phthalate legislation such as the Consumer Product Safety Improvement Act of 2008, which dramatically reduced the amount of six phthalates (including DEHP and DBP) that are permissible in children’s toys. The findings of Swan et al. (2005) have also been discussed and documented in numerous congressional hearings, including the June 2008 House Subcommittee on Commerce, Trade, and Consumer Protection hearing (Committee on Energy and Commerce 2008). This research was also cited heavily in support of regulations passed in California, Vermont, and Washington and introduced in Maine, New York, New Jersey, Connecticut, Maryland, Massachusetts, Rhode Island, West Virginia, Minnesota, Illinois, Oregon, and Hawaii.

The AGD measure developed by Swan et al. (2005) is now being incorporated into multiple ongoing studies. The National Children’s Study (NCS), for example, proposes to include AGD using a similar protocol in the infant exam being piloted in the NCS Vanguard Centers (National Children’s Study 2009). In addition, Swan and colleagues expect funding in mid-2009 for a new multi center pregnancy cohort study that will examine prenatal phthalate exposure in relation to AGD and other reproductive parameters in a larger population using more precise methods. This research will provide standards for measuring genital landmarks obtained in a diverse population-based sample of male infants, which may be suitable for use in pediatric practice. Associations between phthalate metabolites and these androgen-sensitive measures could further impact public health policy, given the ubiquitous nature of these exposures and the clinical importance of appropriate androgen stimulation during fetal development.

## Note from the Editor

### Scientific Integrity

Integrity in science has been a long-time priority for *Environmental Health Perspectives*. Our concern about this issue is reflected in our Instructions to Authors, which outlines our expectations concerning full disclosure of competing financial and nonfinancial interests and potential penalties that could be imposed if journal policy is not followed.

In this issue is an editorial by Jennifer Sass concerning key elements of effective and practical disclosure policies for health science journals. *EHP* staff participated in the development of the document mentioned in the editorial, and we fully endorse recommendations concerning the need for full disclosure of competing interests. As further indication of our commitment to maintaining a high degree of scientific integrity, *EHP* has made the decision to designate a staff member to serve as Ethics Coordinator. The Ethics Coordinator is responsible for ensuring appropriate compliance related to conflict of interest declarations required for each paper, assessing alleged conflicts of interest and plagiarism, and assisting the Editor-in-Chief in assessing potential conflicts of interest of reviewers. Science has made and will continue to make important contributions to the progress of human society. We should, however, be aware that continued support for research is dependent on the perceived integrity of the scientific process. *EHP* remains dedicated to serving as an independent and unbiased venue for the discussion of the impact of the environment on human health.

## Figures and Tables

**Figure:**
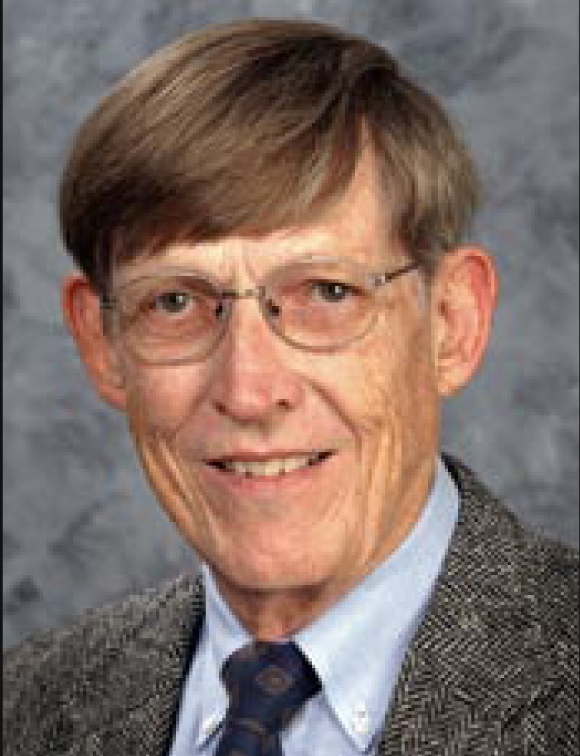
Hugh A. Tilson
